# Screening of *ROS1* Rearrangements in Lung Adenocarcinoma by Immunohistochemistry and Comparison with *ALK* Rearrangements

**DOI:** 10.1371/journal.pone.0103333

**Published:** 2014-07-24

**Authors:** Yoon Jin Cha, Jae Seok Lee, Hye Ryun Kim, Sun Min Lim, Byoung Chul Cho, Chang Young Lee, Hyo Sup Shim

**Affiliations:** 1 Department of Pathology, Severance Hospital, Yonsei University College of Medicine, Seoul, Republic of Korea; 2 Division of Oncology, Department of Internal Medicine, Yonsei Cancer Center, Yonsei University College of Medicine, Seoul, Republic of Korea; 3 Department of Thoracic and Cardiovascular Surgery, Severance Hospital, Yonsei University College of Medicine, Seoul, Republic of Korea; Consiglio Nazionale delle Ricerche (CNR), Italy

## Abstract

*ROS1* rearrangement is a predictive biomarker for response to the tyrosine kinase inhibitor, crizotinib. We investigated the usefulness of ROS1 immunohistochemistry (IHC) for the detection of patients who harbor ROS1 rearrangements in two separate cohorts. We also compared ROS1 IHC with ALK IHC in terms of diagnostic performance to predict each gene rearrangement. In a retrospective cohort, IHC was performed in 219 cases of lung adenocarcinoma with already known genetic alterations. In a prospective cohort, we performed IHC for 111 consecutive cases of lung adenocarcinoma and confirmed the results by subsequent FISH. In the retrospective cohort, all 8 *ROS1*-rearranged tumors were immunoreactive, and 14 of 211 *ROS1*-wild cases were immunoreactive (sensitivity 100% and specificity 93.4%). In the prospective cohort, all IHC-negative cases were FISH-negative, and 5 of 34 ROS1 immunoreactive cases were *ROS1*-rearranged (sensitivity 100% and specificity 72.6%). In *ROS1*-wild tumors, ROS1 protein was more expressed in the tumors of ever-smokers than in those of never-smokers (p = 0.003). ALK IHC showed 100% sensitivity and 98.1 to 100% specificity in both patient cohorts. In conclusion, ROS1 IHC is highly sensitive, but less specific compared with ALK IHC for detection of the corresponding rearrangement. ROS1 IHC-reactive tumors, especially when the tumor is stained with moderate to strong intensity or a diffuse pattern, are recommended to undergo FISH to confirm the gene rearrangement.

## Introduction

Targeted therapies based on molecular diagnostics have opened a new era of personalized medicine in lung cancer treatment [1], [2]. The *EGFR* mutation and *ALK* rearrangement are currently the most important predictive factors for a response to EGFR tyrosine kinase inhibitors (gefitinib, erlotinib, or afatinib) and the ALK inhibitor (crizotinib), respectively [2]. New predictive biomarkers, such as the *ROS1* rearrangement, *RET* rearrangement, *BRAF* mutation, and *HER2* mutation, have emerged in anticipation of personalized therapy based on molecular diagnostics and targeted therapy [3]. Among these, *ROS1* and *ALK* gene fusions are unique in that they are derived from a chromosomal rearrangement, the tyrosine kinase domains are similar to each other, and they are strongly predictive of response to an ALK inhibitor, such as crizotinib [4]. Thus, it is clinically important to detect patients who will benefit from such inhibitor treatment. However, these rearrangements are rare, comprising 2–5% of all non-small cell carcinomas [5]–[7]. Thus, an effective screening test is essential.

Currently, fluorescence in situ hybridization (FISH) is the gold standard method to detect patients harboring the *ALK* rearrangement, and has been used as a confirmatory test in the clinicopathologic studies for ROS1 [6], [8]. However, FISH has several limitations such as high cost, time-consuming, and requiring an expert's reading. In contrast, immunohistochemistry (IHC) is less time consuming, is cost-effective, can be performed on small biopsies, and theoretically, can identify all fusion variants [9], [10].

The role of ALK IHC has been extensively studied [11]–[15]. However, there are a few studies on ROS1 IHC [16]–[19], and there have not been any studies on the combined ROS1 and ALK rearrangements, and comprehensive results on both ROS1 and ALK IHC have not been reported to date.

In this study, we investigated the usefulness of ROS1 IHC for the detection of patients who harbor *ROS1* rearrangements in two separate cohorts. We also compared ROS1 IHC with ALK IHC in terms of diagnostic performance to predict each gene rearrangement.

## Materials and Methods

### Study populations

The study populations were composed of two patient cohorts with histologically confirmed lung adenocarcinoma. The retrospective cohort comprised 219 cases, in which genetic analyses (*EGFR* mutation, *KRAS* mutation, *ALK* rearrangement, and *ROS1* rearrangement) were already performed. This cohort was enriched for tumors from never smokers (178 out of 219; 81.3%). Specimens tested in this cohort were from January 2005 to January 2012 and consisted of 103 small biopsy samples (66 from lung, 6 pleura, 22 lymph node, and 9 soft tissue) and 116 large samples including open resection and excisional biopsy (102 from lung, 1 pleura, 9 lymph node, and 4 brain). The prospective cohort comprised 111 cases that were prospectively examined for the expression of ROS1 and ALK by IHC and confirmed by FISH. Mutation analysis for the *EGFR* and *KRAS* genes was also performed in 82 patients (73.9%) of the prospective cohort. Specimens tested in this cohort were from February 2013 to May 2013 and consisted of 64 small biopsy samples (44 from lung, 3 pleura, 14 lymph node, and 3 soft tissue) and 47 large samples including open resection and excisional biopsy (42 from lung, 4 lymph node, and 1 brain). This study was approved by the Institutional Review Board of Severance Hospital. All patients provided written informed consent for the genetic analysis.

### Histologic review

Samples were classified according to the 2011 International Association for the Study of Lung Cancer/American Thoracic Society/European Respiratory Society guidelines [20]. When poorly differentiated tumors were encountered, immunohistochemistry for TTF-1, napsin A, and p63 (or p40) was performed to differentiate adenocarcinoma from squamous cell carcinoma [21].

### *EGFR* and *KRAS* mutation analysis

To determine the *EGFR* and *KRAS* mutation status, DNA was extracted using a DNeasy isolation kit (Qiagen, Valencia, CA, USA) from formalin-fixed paraffin-embedded tissues according to the manufacturer's instructions. For the *EGFR* gene, direct DNA sequencing of exons 18 through 21 was performed in the retrospective cohort, and the peptide nucleic acid clamping method was performed in the prospective cohort [22]. For the *KRAS* gene, direct DNA sequencing of codons 12 and 13 was performed. Each case was classified as positive or negative for a mutation based on comparison with the wild-type sequence.

### Fluorescence *in situ* hybridization

To identify *ROS1* and *ALK* rearrangements, fluorescent in situ hybridization (FISH) was performed on the whole section of formalin-fixed, paraffin-embedded (FFPE) tumors using a break-apart ROS1 or ALK probe (Vysis LSI Dual Color, Break Apart Rearrangement Probe; Abbott Molecular, Abbot Park, IL, USA), respectively. *ROS1* or *ALK* rearrangements were scored as positive when >15% of tumor cells displayed split signals or isolated signals containing a kinase domain (green for *ROS1* and red for *ALK*), as previously described [6], [23].

### Immunohistochemistry and interpretation

FFPE tissues were sectioned at a thickness of 4 µm and stained using the Ventana automated immunostainer BenchMark XT (Ventana Medical Systems, Tucson, AZ, USA). The slides were dried at 60°C for 1 hour and deparaffinized using EZ Prep (Ventana Medical Systems) at 75°C for 4 minutes. Cell conditioning was performed using CC1 solution (Ventana Medical Systems) at 100°C for 64 minutes. ROS1 (rabbit monoclonal, clone D4D6, Cell Signaling Technology, Danvers, MA, USA) and ALK (rabbit monoclonal, clone D5F3, Cell Signaling Technology, Danvers, MA, USA) antibodies were diluted to 1∶50, treated, and incubated at 37°C for 32 minutes. Signals were detected using the OptiView DAB IHC Detection Kit (Ventana Medical Systems). Counterstaining was performed with Hematoxylin I (Ventana Medical Systems) for 4 minutes at room temperature. IHC-positive controls included gene rearranged lung tumor confirmed by FISH. Negative controls included non-rearranged lung tumor confirmed by FISH as well as normal lung tissue.

The stained slides were reviewed by three pathologists (Y.J.C., J.S.L., and H.S.S.) blinded to FISH results. To analyze the IHC results in detail, the stained slides were scored by the H-score method, which is the sum of products of multiplying intensity (0, 1, 2, and 3) by extent of each staining intensity (%) [16]. H-scores range from 0 to 300. The definition of intensity was as follows: 0 for no detectable staining, 1+ for weak reactivity mainly detectable at high magnification (20–40× objective), and 2+ or 3+ for more intense (moderate or strong, respectively) reactivity easily detectable at low magnification (4× objective) [16]. In cases with a discrepancy in IHC scoring, all pathologists reviewed the cases in conference and a consensus score was established.

### Statistical analysis

Relationships between clinicopathologic parameters were evaluated using the chi-square test. Student's t-test was used to compare means between two groups. Differences were considered significant for p<0.05. All statistical analyses were conducted using SPSS v.17 (SPSS, Chicago, IL).

## Results

### FISH and IHC in the retrospective cohort

A total of 219 cases were evaluated for *ROS1* and *ALK* rearrangements using FISH and subsequent IHC. The clinicopathologic characteristics of the retrospective cohort are summarized in Table 1.

**Table 1:** Clinicopathologic Characteristics of Patients

Factors		Retrospective Cohort (n = 219)	Prospective Cohort (n = 111)
Mean age (range)		59.2 (28–86)	63.9 (32–84)
Sex	Male	68 (31.1)	68 (61.3)
	Female	151 (68.9)	43 (38.7)
Smoking status	Never	178 (81.3)	55 (49.5)
	Former	17 (7.8)	33 (29.7)
	Current	24 (11.0)	23 (20.7)
Stage	I	38 (17.4)	22 (19.8)
	II	23 (10.5)	9 (8.1)
	III	60 (27.4)	19 (17.1)
	IV	98 (44.7)	61 (55.0)
Mutation status	EGFR	68 (31.1)	31 (27.9)
	KRAS	10 (4.6)	9 (8.1)
	ROS1	8 (3.7)	5 (4.5)
	ALK	12 (5.5)	8 (7.2)
	Pan-negative	121 (55.3)	29 (26.1)
	Not available	0 (0.0)	29 (26.1)

In FISH analyses, 8 of 219 (3.7%) were *ROS1*-rearranged, and 12 of 219 (5.5%) tumors were *ALK*-rearranged by FISH. These rearrangements were mutually exclusive.

**Figure 1:**
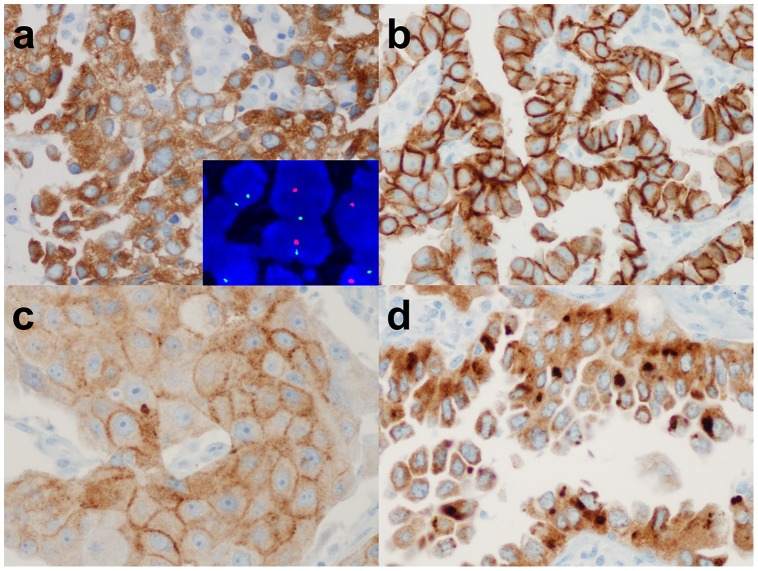
Immunohistochemical findings of *ROS1*-rearranged tumors. An *ROS1*-rearrangned tumor showed strong and diffuse cytoplasmic staining (a) (inset: *ROS1* FISH with split signals). Other tumors showed cytoplasmic staining with membrane accentuation (b), membranous staining with weaker cytoplasmic intensity (c), or cytoplasmic or paranuclear aggregates (d).

**Figure 2:**
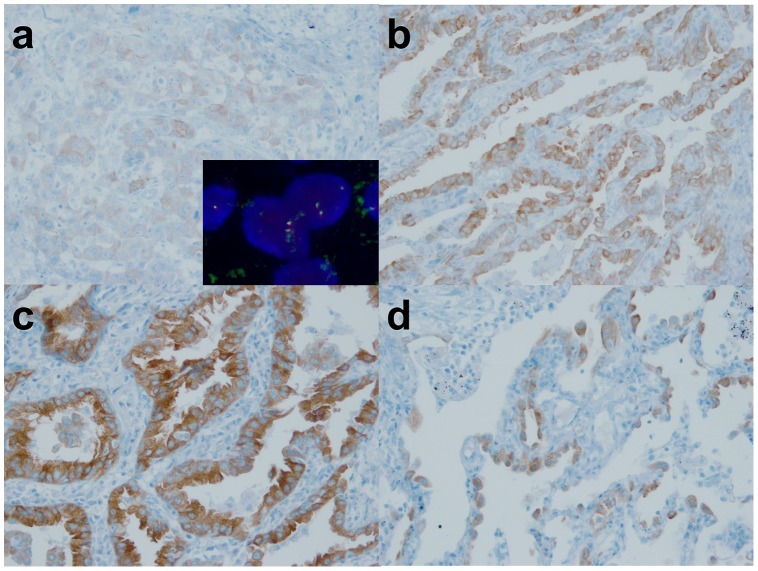
Immunohistochemical findings of *ROS1*-non-rearranged tumors. Most *ROS1*-non-rearranged tumors showed no immunoreactivity, or focal and patchy staining with weak intensity (a, b) (inset: *ROS1* FISH without split signals). One tumor showed strong and diffuse staining pattern, which is similar to that of rearranged tumors (c). ROS1 is occasionally expressed in surrounding type II pneumocytes (d).

**Figure 3:**
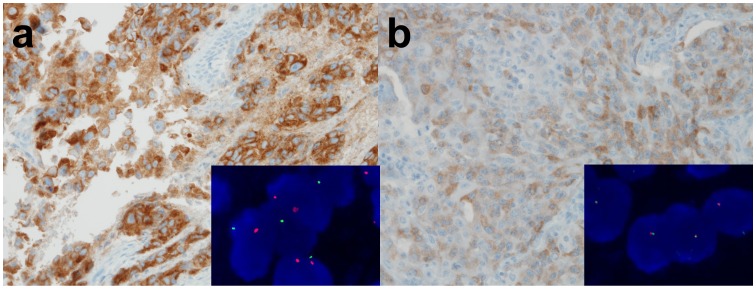
Representative photos from ALK IHC. An *ALK*-rearranged tumor shows strong and diffuse staining (a) (inset: *ALK* FISH with split signals). In contrast, an *ALK*-non-rearranged tumor shows weak and patchy staining (b) (inset: *ALK* FISH without split signals).

**Figure 4:**
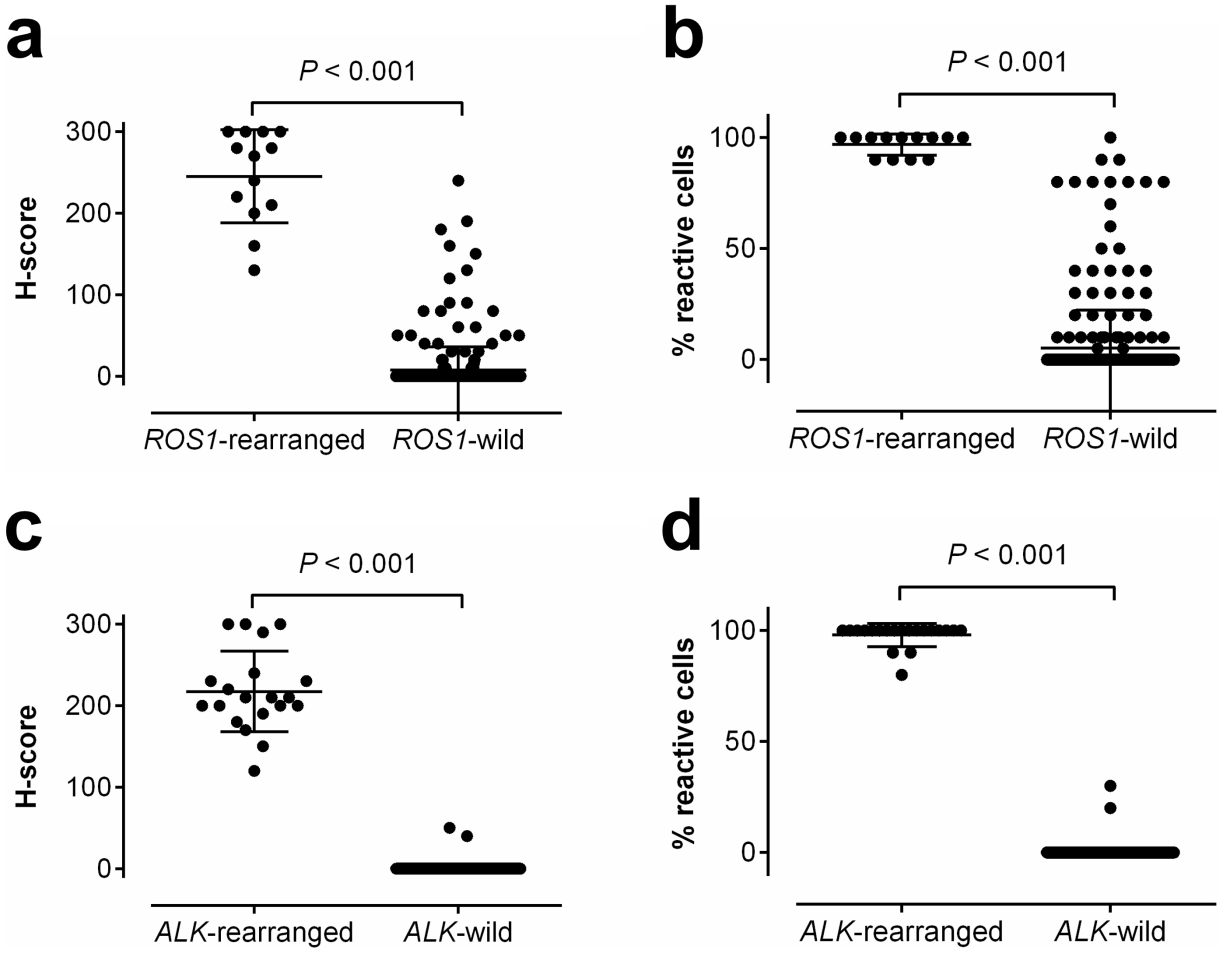
Comparison of the H-score and extent of ROS1 (a, b) and ALK (c, d) immunoreactivity in gene-rearranged *vs.* non-rearranged tumors. Scatter dot plots for H-scores (a, c) and percentage of immunoreactive cells (b, d) show significantly increased expression of each protein in rearranged tumors (p<0.001). All rearranged tumors show an H-score of more than 100 and extent of more than 75%.

In IHC analyses, all 8 *ROS1*-rearranged cases were ROS1-immunoreactive, showing an H-score of more than 100, extent of more than 75%, and the presence of 2+ or 3+ intensity (Figure 1). Of the 211 *ROS1*-wild cases, 14 cases were also ROS1-immunoreactive (Figure 2). However, most cases showed focal and weak immunoreactivity (H-score mean: 46.4, range: 5–160). All 12 *ALK*-rearranged tumors were ALK immunoreactive, showing an H-score of more than 100, an extent of more than 75%, and the presence of 2+ or 3+ intensity (Figure 3a). In contrast, all *ALK*-wild cases showed no immunoreactivity. Both the H-score and extent were significantly different between *ROS1*- or *ALK*-rearranged and wild groups (Figure 4, p<0.001). There were no double-immunoreactive cases for both ROS1 and ALK IHC. In 2 cases, the surrounding reactive type II pneumocytes showed ROS1 immunoreactivity (Figure 2d).

### IHC and FISH in the prospective cohort

A total of 111 cases with unknown genetic alterations were screened using ROS1 and ALK IHC, and all cases were evaluated for *ROS1* and *ALK* rearrangements using FISH. The clinicopathologic characteristics of the prospective cohort are summarized in Table 1. In IHC analyses, 34 of 111 (30.6%) were ROS1 immunoreactive, and 10 of 111 (9.0%) tumors were ALK immunoreactive. In subsequent FISH analyses, 5 of 34 ROS1 immunoreactive tumors were *ROS1*-rearranged by FISH. The IHC of all 5 *ROS1*-rearranged tumors showed an H-score of more than 100, an extent of more than 75%, and the presence of 2+ or 3+ intensity (Figures 1 and 4). Twenty-nine IHC-reactive/FISH-negative cases showed variable H-scores (mean: 58.8; range 10–240), extent (mean: 40; range 10 to 100), and intensity (1+ to 3+) (Figures 2 and 4). All ROS1 IHC-negative tumors were *ROS1*-wild by FISH.

Regarding ALK, 8 of 10 immunoreactive tumors were *ALK*-rearranged. The IHC of all 8 *ALK*-rearranged tumors showed an H-score of more than 100, an extent of more than 75%, and the presence of 2+ or 3+ intensity. Two cases that were IHC-reactive/FISH-negative showed less than 50% extent and an H-score of less than 100 (Figure 3b). All ALK IHC-negative tumors were *ALK*-wild by FISH. Both the H-score and extent were significantly different between the *ROS1*- or *ALK*-rearranged and -wild groups (Figure 4, p<0.001).

There were 2 double-immunoreactive cases for ROS1 and ALK IHC. For the first case, the H-scores were 30 and 190 for ROS1 and ALK, respectively, and this case was *ALK*-rearranged by FISH. The second case's H-scores were 20 and 40 for ROS1 and ALK, respectively, and was wild-type for both genes by FISH. As in the retrospective cohort, the surrounding reactive type II pneumocytes were ROS1-immunoreactive in 6 cases.

### IHC criteria to predict gene rearrangements

In the retrospective cohort, any immunoreactivity for ROS1 showed 100% sensitivity and 93.4% specificity to predict a *ROS1* rearrangement. When the criteria were defined as (1) H-score of 100 or more, or (2) extent of 75% or more, or (3) presence of 2+ or 3+ intensity, the specificities were (1) 99.1%, (2) 98.6%, or (3) 98.1%, respectively. All criteria maintained 100% sensitivity. In the prospective cohort, these criteria showed 100% sensitivity, but the specificities were (1) 95.3%, (2) 93.4%, or (3) 88.7%, respectively. In both patient cohorts, these criteria showed 100% sensitivity and 95 to 97.8% specificity (Table 2).

**Table 2:** Diagnostic performance of IHC to predict gene rearrangement according to cutoff in both patient cohorts

		ROS1		ALK	
Cutoff		Sensitivity	Specificity	Sensitivity	Specificity
H-score	>0	100%	86.4%	100%	99.4%
	≥50	100%	94.3%	100%	99.7%
	**≥100**	**100%**	**97.8%**	**100%**	**100%**
	≥150	92.3%	98.4%	95.0%	100%
	≥200	84.6%	99.7%	75.0%	100%
	≥250	53.8%	100%	20.0%	100%
Extent	≥25%	100%	92.4%	100%	99.7%
	≥50%	100%	95.6%	100%	100%
	**≥75%**	**100%**	**96.8%**	**100%**	**100%**
Intensity	**≥2+**	**100%**	**95.0%**	**100%**	**99.4%**
	= 3+	84.6%	98.4%	75%	100%

Regarding ALK in the retrospective cohort, any immunoreactivity showed 100% sensitivity and 100% specificity to predict an *ALK* rearrangement. In the prospective cohort, ALK IHC was also highly sensitive and specific (sensitivity 100% and specificity 98.1%). When the criteria were defined as H-score of 100 or more, or extent with 75% or more, the sensitivity and specificity were 100% in both patient cohorts (Table 2).

### ROS1 or ALK expression in non-rearranged tumors from both patient cohorts

**Figure 5:**
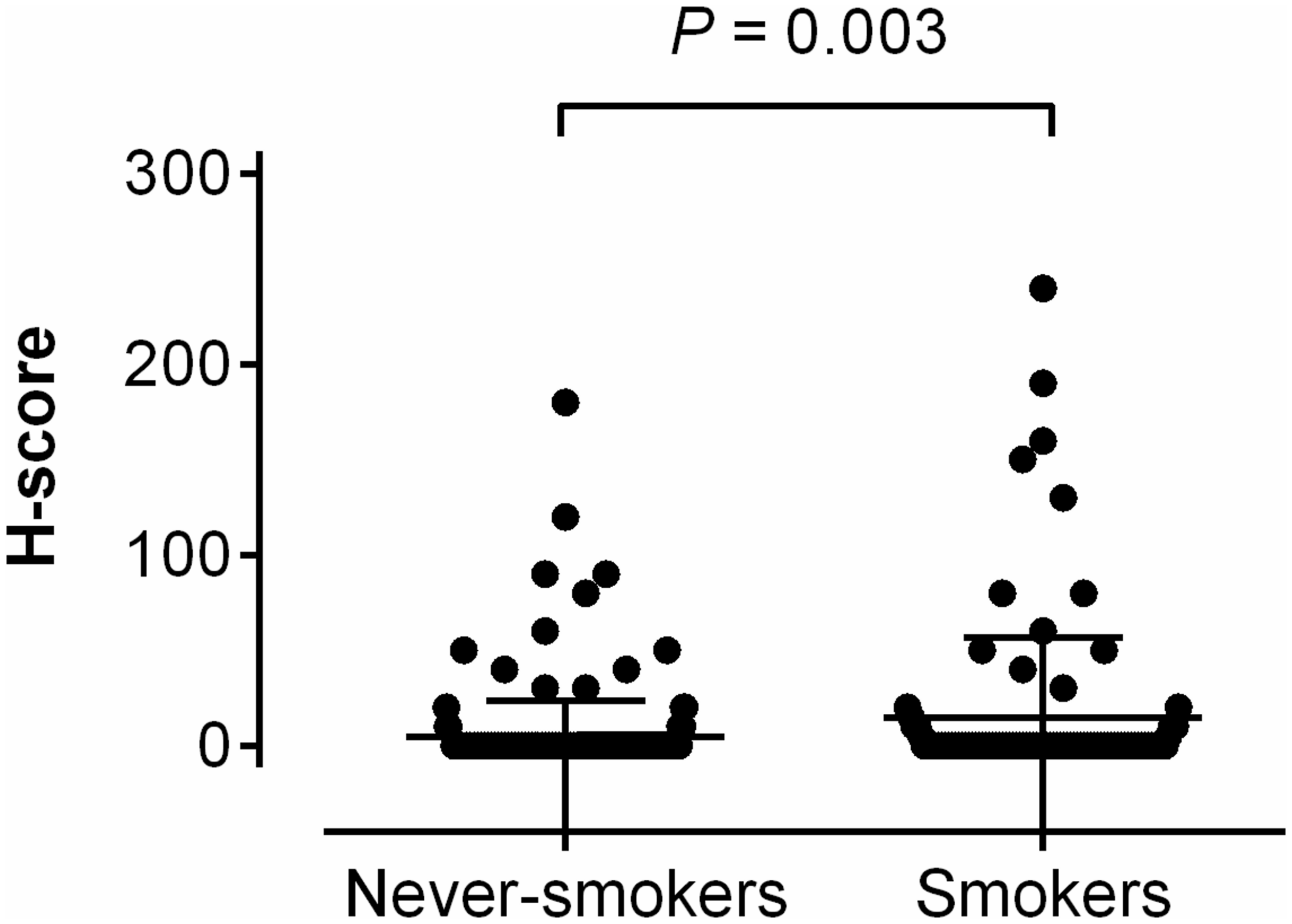
Comparison of the H-score in ROS1-non-rearranged tumors according to smoking history. The scatter dot plot shows that the ROS1 protein is more expressed in tumors of smokers' than in those of never-smokers (p = 0.003).

ROS1 protein was expressed in 43 of 317 ROS1-non-rearranged tumors (13.6%) (Figure 2). The staining was sometimes diffuse or intense (mean of H-score: 54.8, range: 5–240) (Figure 2c). ROS1 protein was more expressed in the tumors of ever-smokers than in those of never-smokers (Figure 5, p = 0.003). In contrast, ALK protein was expressed in only 2 cases of 310 ALK-non-rearranged tumors (0.6%) (Figure 3b). The staining was focal and weak (H-score: 40 and 50), and both tumors were from never-smokers.

### Clinical and pathologic characteristics in both patient cohorts

Patients with *ROS1* or *ALK* rearrangements were significantly younger than those with wild-type genes, as previously known (Table 3). Never-smokers occupied 69.2% and 75.0% of patients with *ROS1* and *ALK* rearrangements, respectively (Tables 3, and Table S1 and S2). However, clinical factors did not perfectly predict which patient's tumor harbors gene rearrangements. Regarding drivers of genetic alterations, the *EGFR* mutation, *KRAS* mutation, *ROS1* rearrangement, and *ALK* rearrangement were mutually exclusive in both cohorts.

**Table 3:** Clinicopathologic Characteristics of *ROS1* and *ALK*-rearranged adenocarcinomas

Factors		All patients (n = 330)	*ROS1*-positive (n = 13)	*ALK*-positive (n = 20)	WT/WT (n = 297)	p-value (*ROS1* vs WT/WT)	p-value (*ALK* vs WT/WT)
Mean age (range)		60.8 (28–86)	54.5 (30–84)	51.1 (28–72)	61.7 (29–86)	0.023	<0.001
Sex	Male	136 (41.2)	6 (46.2)	7 (35.0)	123 (41.4)	0.734	0.572
	Female	194 (58.8)	7 (53.8)	13 (65.0)	174 (58.6)		
Smoking status	Never	233 (70.6)	9 (69.2)	15 (75.0)	209 (70.4)	0.660	0.389
	Former	50 (15.2)	3 (23.1)	1 (5.0)	46 (15.5)		
	Current	47 (14.2)	1 (7.7)	4 (20.0)	42 (14.1)		
Stage	I	60 (18.2)	2 (15.4)	1 (5.0)	57 (19.2)	0.247	0.151
	II	32 (9.7)	0 (0.0)	4 (20.0)	28 (9.4)		
	III	79 (23.9)	6 (46.2)	3 (15.0)	70 (23.6)		
	IV	159 (48.2)	5 (38.5)	12 (60.0)	142 (47.8)		

Four predominant histologic types except lepidic were observed in gene-rearranged cases, although comprehensive analyses were limited by presence of small biopsies (Table S1 and S2). *ROS1*-rearranged tumors showed a solid-predominant pattern (7.7%), cribriform with extracellular mucin (15.4%), and the presence of signet ring cells (7.7%) (Table S1). *ALK* rearranged tumors also showed a solid-predominant pattern (50%), cribriform with extracellular mucin (25%), and the presence of signet ring cells (45%) (Table S2). However, these incidences were more frequent than those of *ROS1*-rearranged tumors.

## Discussion

In the present study, we demonstrated that IHC is an effective screening tool for the detection of *ROS1* rearrangements in lung adenocarcinomas. We also showed that IHC is a sensitive tool, but that non-rearranged tumors can be immunoreactive, so confirmatory FISH is necessary, especially in ROS1-expressed tumors.

The *ALK* rearrangements have been the focus of intense research over the last several years [2], [5], [8], [9], [11]–[15], [23], [24]. Specifically, there have been many studies showing that IHC is a good prescreening method for the detection of patients with *ALK* rearrangement [9], [11]–[15], [25]. IHC is cost-effective and available in most pathology laboratories. In addition, because most lung cancer patients present with advanced-stage disease at the time of diagnosis, the diagnosis of lung cancer is often based on only a small biopsy. IHC can be performed on a few cancer cells. To use IHC as a screening test in molecular-based targeted therapy, its validation is necessary including correlation with the results of other molecular methods. In our study, all FISH-positive cases were immunoreactive, which was consistent with most previous reports. Studies concerning ROS1 IHC also have been reported recently [16], [17], [19]. ROS1 IHC showed 100% sensitivity for the detection of *ROS1* rearrangements in our study.

However, the specificity was different. While ALK IHC showed 99.4% specificity in all patient cohorts, ROS1 IHC showed 86.4% specificity. Remarkably, specificity was the lowest in the prospective cohort (72.6%). This indicated that the expression of ALK is very rare in *ALK*-non-rearranged tumors, but ROS1 is occasionally expressed in *ROS1*-non-rearranged tumors. Interestingly, when confined to cases with *ROS1*-wild alone, ROS1 was more expressed in smokers' tumors (Figure 5). This result was also consistent with the notion that ROS1 immunoreactivity was more prevalent in the prospective cohort, of which ever-smokers comprised 50.5% (compared with 18.7% in the retrospective cohort). Further studies are necessary to elucidate whether ROS1 is also implicated in tumor progression of some *ROS1*-non-rearranged tumors and whether its expression could be affected by smoking.

**Figure 6:**
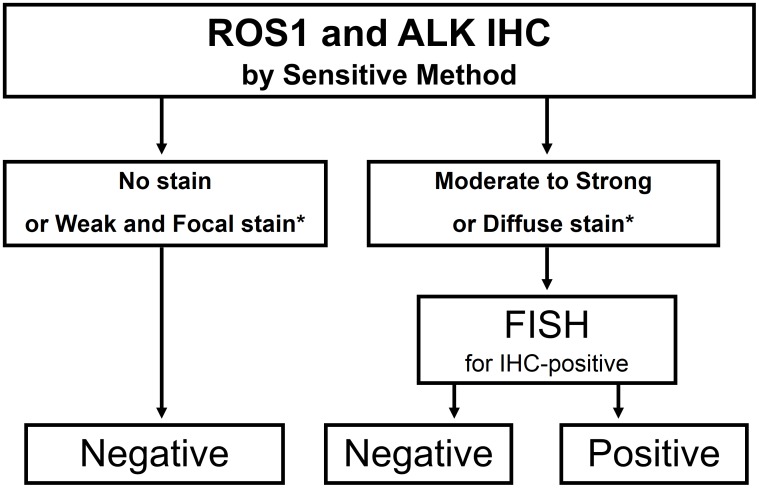
Proposed diagnostic algorithm using ROS1 and ALK IHC. When the tumor shows moderate to strong, or diffuse staining on IHC, FISH is recommend to confirm gene rearrangements. *The criteria should be determined based on each institutional method. According to this present study, subsequent FISH analysis is recommended for cases with an H-score ≥100, extent ≥75%, or the presence of intensity 2+ or 3+.

This present study also confirmed that, although ROS1 or ALK proteins can be expressed in non-rearranged tumors, most cases showed focal extent or a weak intensity pattern. Thus, we could determine the IHC cutoff to predict gene rearrangement more specifically (Table 2). In our study, all FISH-positive cases showed an H-score of 100 or more, extent of 75% or more, or the presence of 2+ or 3+ intensity on IHC. These cutoff lines showed 95 to 100% specificity in both patient cohorts, maintaining 100% sensitivity. Thus, we propose the diagnostic algorithm shown in Figure 6. Because gene rearrangements are rare, sensitive IHC screening is reasonable before FISH. In addition, a proper cutoff can enrich FISH-positive cases.

Our study indicated that a small biopsy sample from patients with advanced stage can be enough and representative for IHC screening. We tested it through the prospective cohort. All FISH-positive cases showed diffuse staining pattern including biopsy samples. For rearranged tumors, heterogeneous staining pattern, that is definitive positive in one area and definitive negative in other area, was very rare.

**Figure 7:**
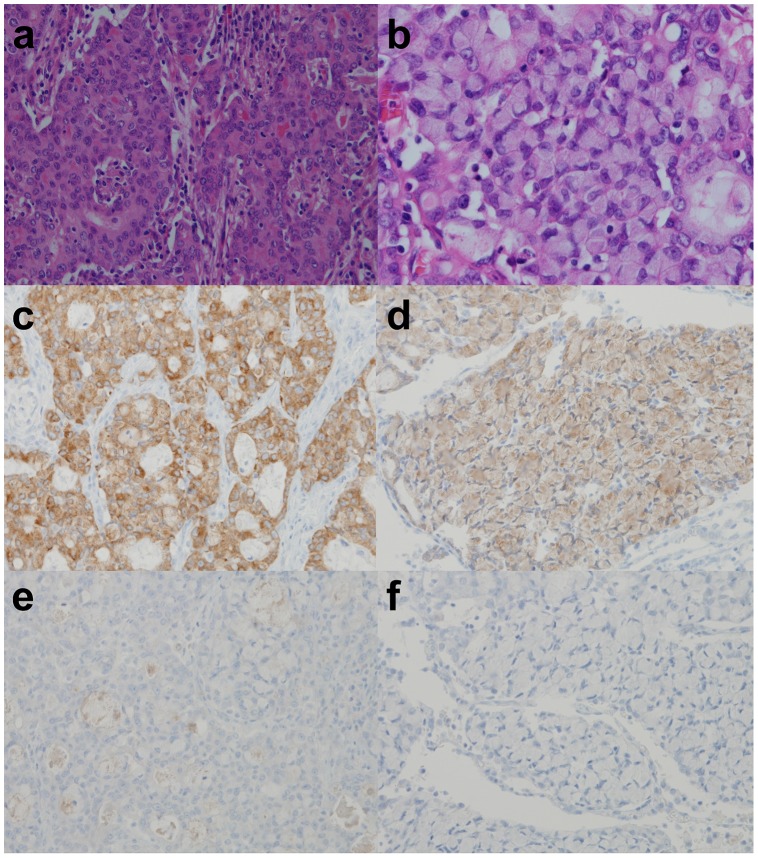
Heterogeneity of histology and immunohistochemical staining. One area of a *ROS1*-rearranged tumor shows a solid area with intense immunostaining for ROS1 (a and c). Another area shows a predominantly signet ring cell feature with weaker immunostaining (b and d). Both areas were ALK IHC-negative (e and f).

As shown in previous studies, ROS1 or ALK IHC showed a predominantly cytoplasmic staining pattern. However, some cases showed a membrane-accentuated pattern, or a cytoplasmic or paranuclear aggregated pattern, especially in ROS1-rearranged tumors (Figure 2). These patterns were reported to be associated with molecular variants [16], [18]. Most rearranged tumors showed an intense staining pattern, but in the signet ring cell areas, the expression was weaker (Figure 7), as noted in previous reports [11], [16].

Our study also suggested that *ROS1*- or *ALK*-rearranged tumors are related to some clinicopathologic features, such as young age, never-smokers, and histology with a mucinous cribriform pattern or signet ring cells, as shown in previous reports [24], [26]. However these characteristics were not perfectly correlated with the presence of gene rearrangements. Given that driver genetic alterations (*EGFR* mutation, *KRAS* mutation, *ROS1* rearrangement, and *ALK* rearrangement) were mutually exclusive in both cohorts, driver mutation-negative cases are highly recommended for further genetic testing. Specifically, the combined incidence of *ROS1* and *ALK* rearrangements increased from 10% in all patients to 15.6% in patients with wild-type *EGFR* and *KRAS*.

In conclusion, IHC is an effective screening tool for the detection of *ROS1*-rearranged adenocarcinomas. ROS1 IHC is highly sensitive, but less specific compared with ALK IHC in that ROS1 is more frequently expressed in non-rearranged tumors. ROS1 IHC-reactive tumors, especially when the tumor is stained with moderate to strong intensity or a diffuse pattern, are recommended to undergo FISH to confirm the gene rearrangement.

## Supporting Information

Table S1 **Clinicopathologic details of patients with** ***ROS1*** **-rearranged adenocarcinoma.**
(DOC)

Table S2 **Clinicopathologic details of patients with** ***ALK*** **-rearranged adenocarcinoma.**
(DOC)
