# Supplementary material for: Screening of ROS1 Rearrangements in Lung Adenocarcinoma by Immunohistochemistry and Comparison with ALK Rearrangements
Source: PLoS One. 2014 Jul 24;9(7):e103333. doi: 10.1371/journal.pone.0103333 (PMC4109990; doi:10.1371/journal.pone.0103333)
Supplement: Table S1 — Clinicopathologic details of patients with ROS1 -rearranged adenocarcinoma. (DOC) [file pone.0103333.s001.doc]

**Supporting Information**

Table S1. Clinicopathologic details of patients with *ROS1*-rearranged adenocarcinoma

| Patient | Age | Sex | Pack years | Stage | Specimen type | Predominant Pattern | Cribriform pattern with Mucin | Signet ring cells |
| --- | --- | --- | --- | --- | --- | --- | --- | --- |
| 1 | 55 | F | 0 | IIIa | Lung resection | Acinar | No | No |
| 2 | 67 | F | 0 | IV | Lymph node biopsy | Micropapillary | No | No |
| 3 | 52 | M | 0 | Ib | Lung resection | Acinar-Cribriform | Yes | No |
| 4 | 30 | F | 0 | IIIa | Lung biopsy | Micropapillary | No | No |
| 5 | 60 | F | 0 | IIIa | Lung biopsy | Acinar | No | No |
| 6 | 60 | F | 0 | IIIb | Lymph node biopsy | Solid | No | No |
| 7 | 84 | M | 44 | IIIb | Lung biopsy | Acinar | No | No |
| 8 | 58 | M | 16 | IV | Lymph node biopsy | Micropapillary | No | No |
| 9 | 59 | F | 0 | IV | Lung biopsy | Acinar | No | No |
| 10 | 32 | F | 0 | IV | Lymph node biopsy | Acinar | No | No |
| 11 | 62 | M | 0 | Ia | Lung resection | Acinar | No | No |
| 12 | 39 | M | 11 | IV | Lymph node biopsy | Papillary | No | No |
| 13 | 51 | M | 10 | IIIa | Lung resection | Acinar-Cribriform | Yes | > 10% |
