# Supplementary material for: Screening of ROS1 Rearrangements in Lung Adenocarcinoma by Immunohistochemistry and Comparison with ALK Rearrangements
Source: PLoS One. 2014 Jul 24;9(7):e103333. doi: 10.1371/journal.pone.0103333 (PMC4109990; doi:10.1371/journal.pone.0103333)
Supplement: Table S2 — Clinicopathologic details of patients with ALK -rearranged adenocarcinoma. (DOC) [file pone.0103333.s002.doc]

**Supporting Information**

Table S2. Clinicopathologic details of patients with *ALK*-rearranged adenocarcinoma

| Patient | Age | Sex | Pack years | Stage | Specimen type | Predominant Pattern | Cribriform pattern with Mucin | Signet ring cells |
| --- | --- | --- | --- | --- | --- | --- | --- | --- |
| 1 | 28 | F | 0 | IIa | Lung resection | Acinar-Cribriform | Yes | ≥ 10% |
| 2 | 49 | F | 0 | IIa | Lung resection | Solid | No | < 10% |
| 3 | 55 | M | 0 | IIa | Lung resection | Acinar | No | No |
| 4 | 37 | F | 0 | IV | Lymph node biopsy | Acinar-Cribriform | Yes | No |
| 5 | 64 | F | 0 | IV | Lung biopsy | Solid | No | ≥ 10% |
| 6 | 57 | F | 0 | IV | Lung biopsy | Acinar | No | No |
| 7 | 64 | F | 0 | IV | Soft tissue biopsy | Solid | No | No |
| 8 | 34 | F | 0 | IV | Lung resection | Solid | Yes | ≥ 10% |
| 9 | 72 | F | 0 | IV | Lung biopsy | Micropapillary | No | No |
| 10 | 62 | M | 40 | IV | Lymph node biopsy | Micropapillary | No | No |
| 11 | 48 | M | 13 | IV | Lung biopsy | Solid | No | No |
| 12 | 41 | M | 0 | IV | Soft tissue biopsy | Solid | No | No |
| 13 | 45 | F | 0 | IV | Lung biopsy | Micropapillary | No | No |
| 14 | 53 | F | 0 | IV | Lung biopsy | Solid | No | ≥ 10% |
| 15 | 63 | M | 40 | IIIa | Lung resection | Solid | No | ≥ 10% |
| 16 | 68 | F | 0 | IV | Lung biopsy | Acinar | No | No |
| 17 | 42 | M | 30 | IV | Lymph node biopsy | Acinar | No | No |
| 18 | 45 | M | 30 | IIa | Lung resection | Solid | Yes | ≥ 10% |
| 19 | 41 | F | 0 | Ia | Lung resection | Acinar-Cribriform | Yes | <10% |
| 20 | 53 | F | 0 | IIIa | Lung resection | Solid | No | ≥ 10% |
